# The Development of Psoriasis Over a Silicone Granuloma: Case Report and Postulated Mechanism of Pathogenesis

**DOI:** 10.7759/cureus.2684

**Published:** 2018-05-24

**Authors:** Monica Rosales Santillan, Sirunya Silapunt, Jaime A Tschen, Philip R Cohen

**Affiliations:** 1 University of Texas Mcgovern Medical School at Houston, Houston, USA; 2 Dermatology, University of Texas Mcgovern Medical School at Houston, Houston, USA; 3 Dermpath, St Joseph Dermpath, Houston, USA; 4 Dermatologist, San Diego Family Dermatology, National City, USA

**Keywords:** silicone granuloma, psoriasiform, immunocompromised cutaneous district

## Abstract

Liquid silicone injections are used for soft tissue augmentation and have the potential to cause adverse effects. A 60-year-old woman who developed a psoriatic lesion over a silicone granuloma is reported. The clinical, dermatoscopic, and histological findings were characteristic of psoriasis. The patient was started on hydroxychloroquine for the treatment of the granuloma without any subsequent development of new psoriatic lesions. The presentation of psoriasis associated with a silicone granuloma in an immunocompetent patient is unique. The sequence of events is another example of a dermatosis occurring in an immunocompromised cutaneous district.

## Introduction

Silicone is used for soft tissue enhancement. It can cause various adverse immunologic and local effects, such as cellulitis and granulomas. Indeed, adverse effects from liquid silicone injections have been reported several years after the procedure [1-3]. A woman who developed psoriasis of the skin overlying a granuloma on her gluteal cleft following liquid silicone injection is described and a potential mechanism of pathogenesis is postulated.

## Case presentation

A 60-year-old woman presented for evaluation of a pruritic plaque located on the sacral region that had been present for a year. She had received silicone oil injections into the site two years prior to developing the new skin lesion. Neither she nor her family had psoriasis.

**Figure 1:**
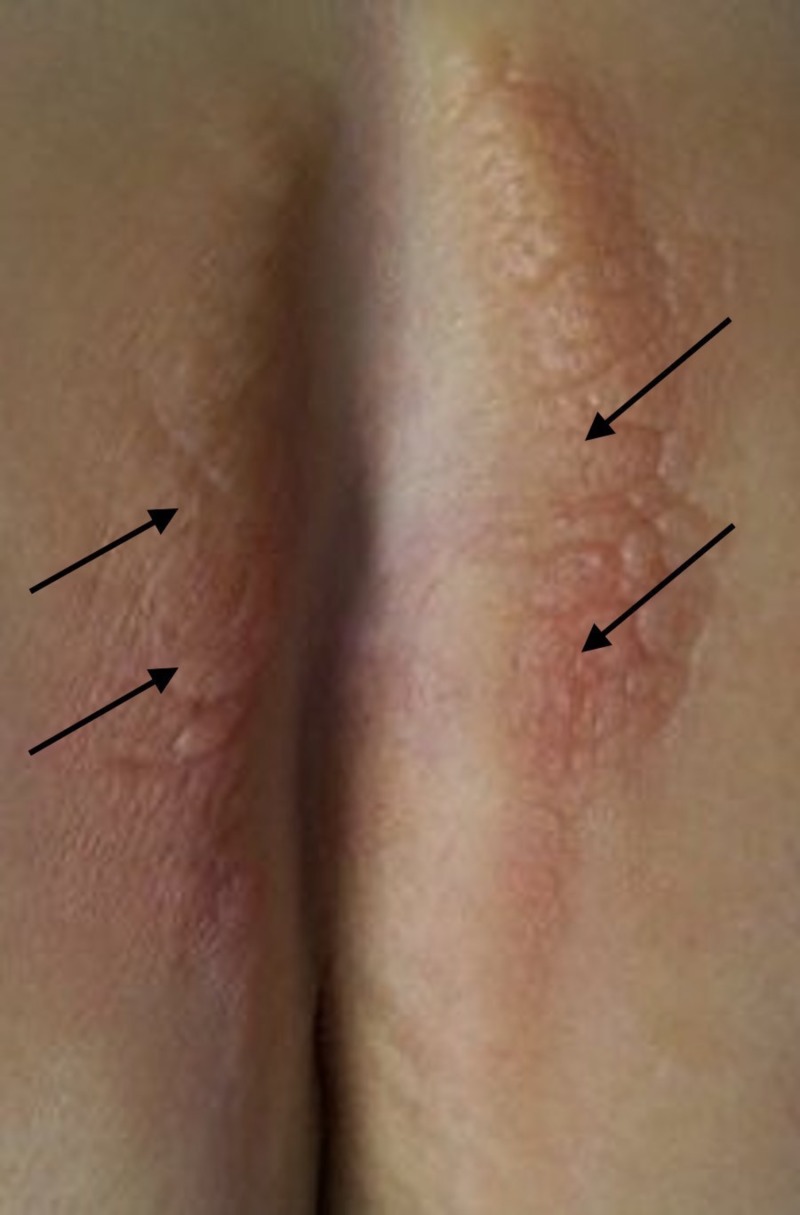
Granulomatous reaction on the buttocks at the site of the silicone injections Yellowish verrucous plaque (arrows) located on the gluteal clefts of both buttocks two years after silicone injection into the areas.

Cutaneous examination revealed yellowish firm verrucous plaques bilaterally located on the skin of her gluteal cleft (Figure 1). The plaques were considered to be a lichenoid granulomatous reaction to the silicone. The patient received four monthly sessions of intralesional triamcinolone acetonide (2 ml of 10 mg/mL); she also used a high-potency topical corticosteroid (clobetasol, 0.05% ointment) once a day for four months, which provided no improvement.

**Figure 2:**
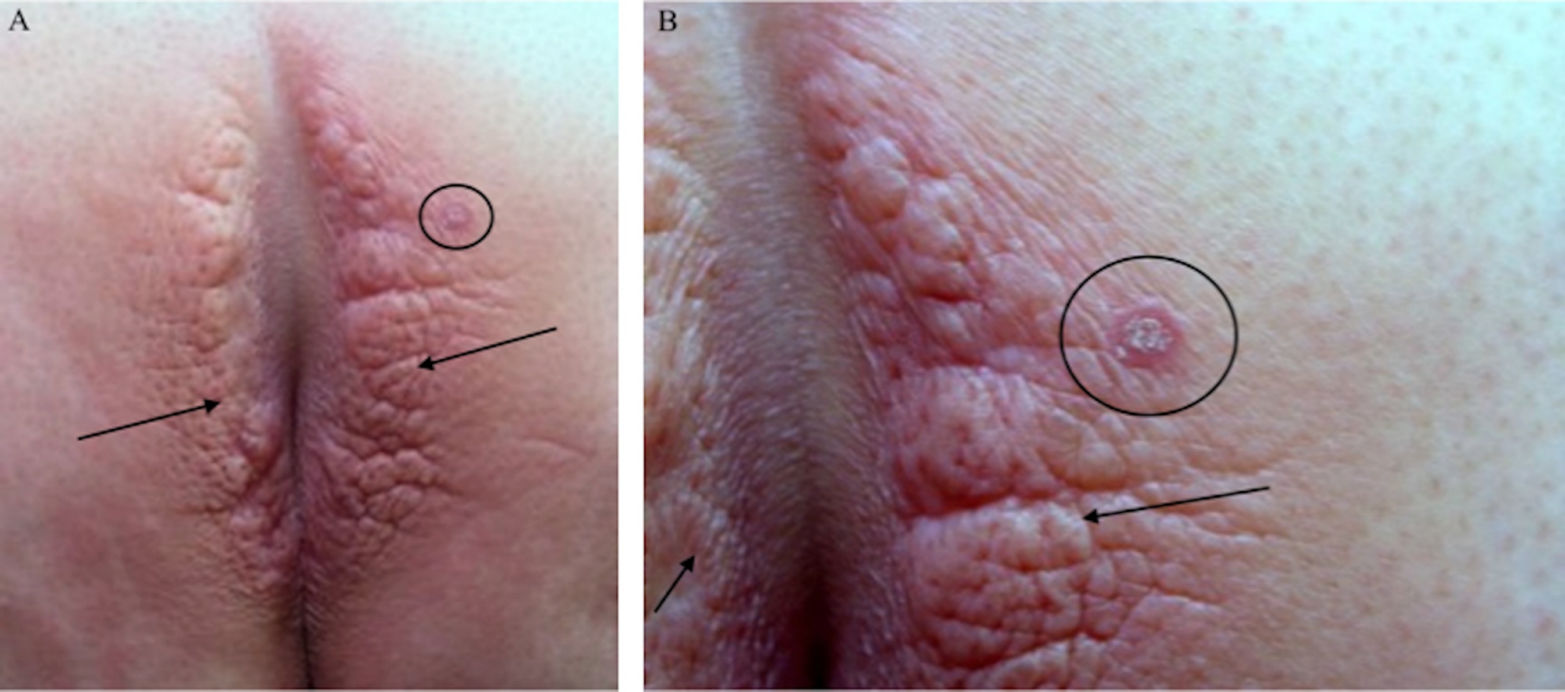
Psoriasis plaque on the right gluteal cleft Distant (A) and closer (B) views six months after the final injection of triamcinolone acetonide. The original plaques appear red (arrows) and a new 6-mm plaque with silvery scale (circle) on the right side of the gluteal cleft has developed.

**Figure 3:**
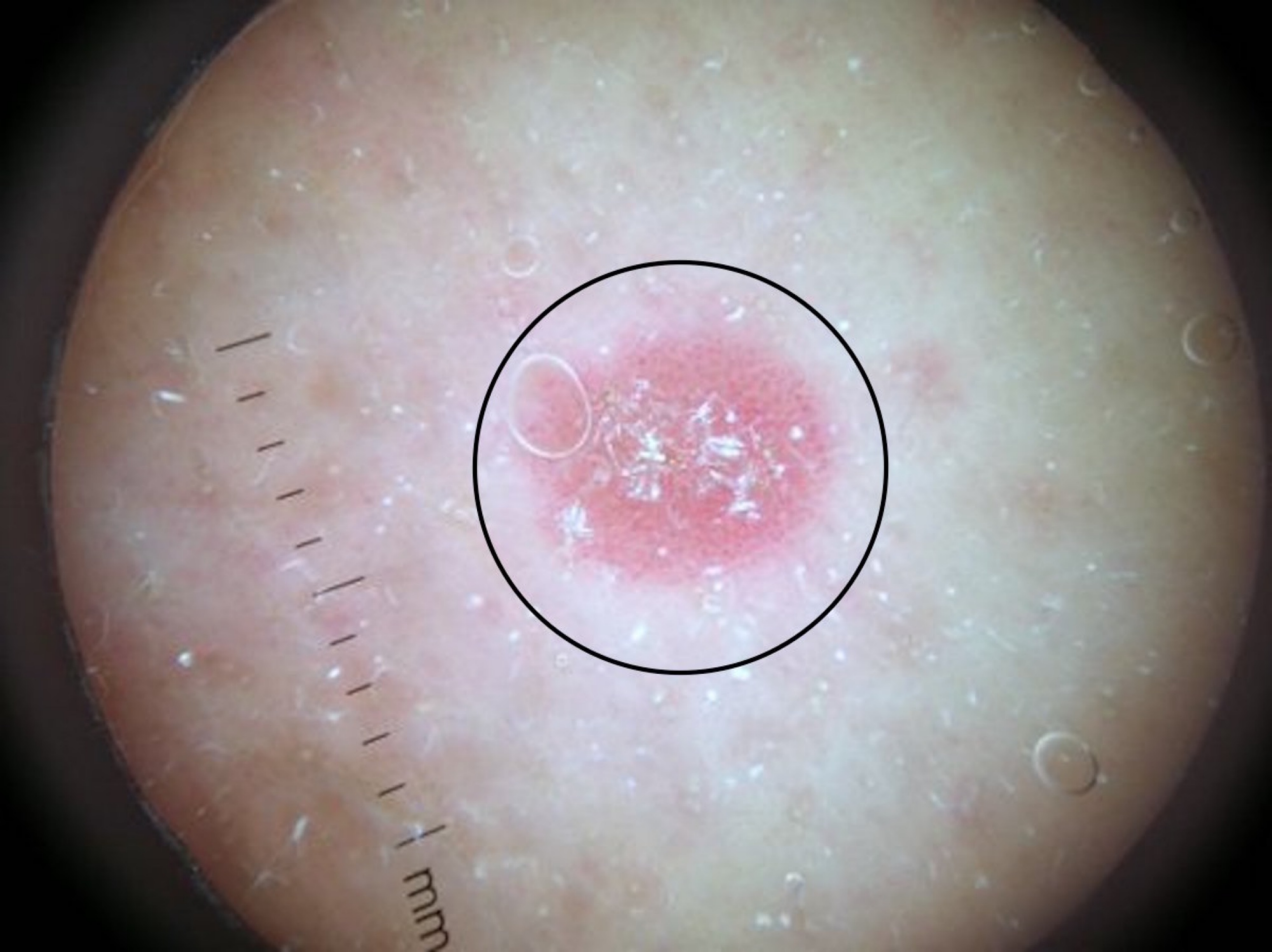
Dermatoscopic image of the psoriasis plaque The psoriasis plaque (circle) demonstrates a scaly surface with red globules characteristic of the blood vessels in the papillary dermis of psoriasis when the lesion is examined with a dermatoscope.

The initial plaques became red and a new 6-mm, erythematous plaque with a silvery scale developed on her right buttock (Figure 2). The morphology of the new right buttock plaque was different than that of her presenting skin lesions; the clinical differential diagnosis of the new plaque included squamous cell carcinoma, psoriasis, and lichen planus. Dermatoscopic evaluation of the new plaque showed a scaly surface with red globules characteristic of the papillary blood vessels observed in psoriasis (Figure 3).

**Figure 4:**
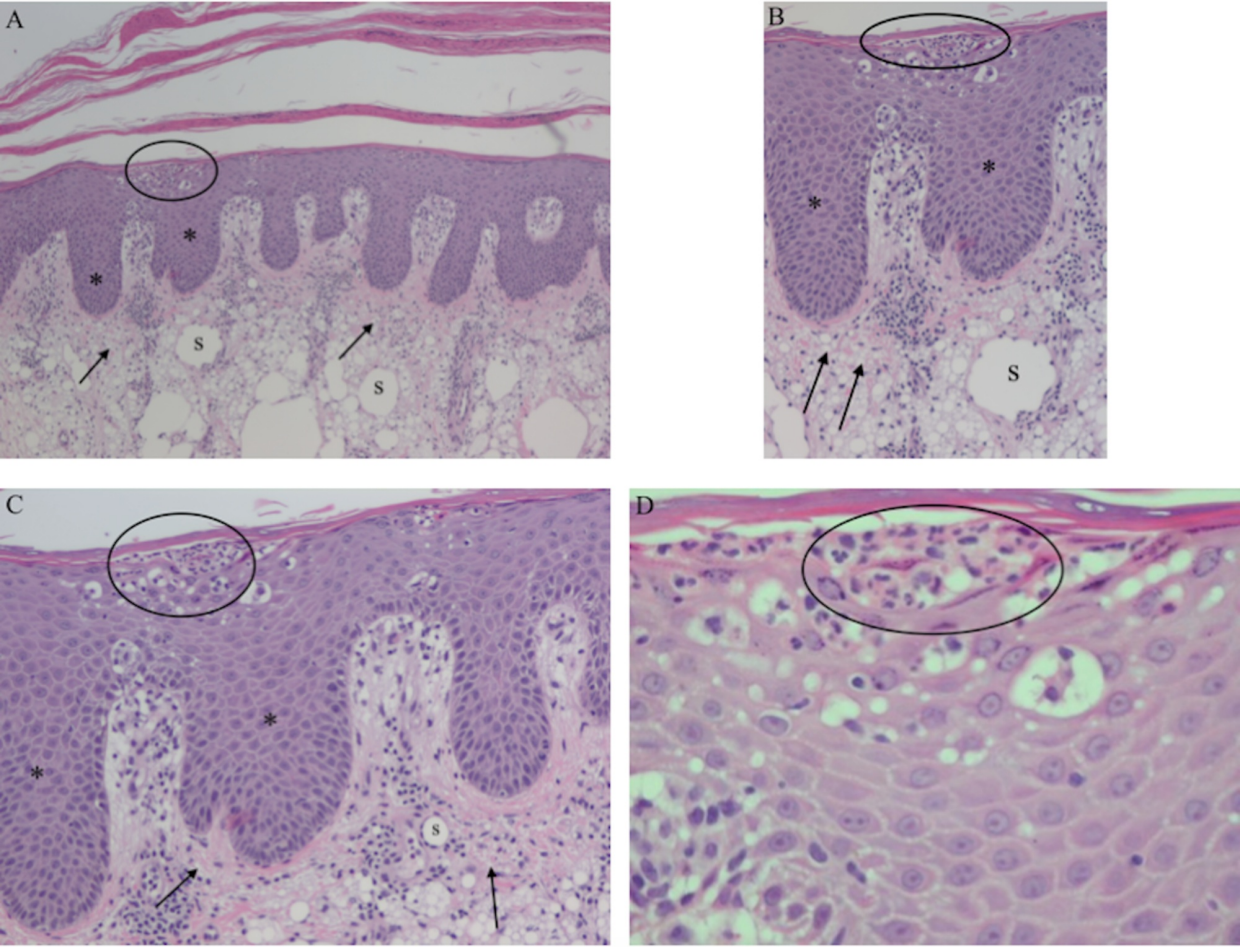
Pathology of psoriasis plaque and underlying silicone granuloma Distant (A) and closer (B, C, and D) views of the skin biopsy pathology. The epidermis shows confluent parakeratosis, regular acanthosis, elongated rete ridges (asterisk), and a thin or absent granular layer; in addition, neutrophilic microabscesses are present (circle) in the upper layers of the epidermis. The upper dermis shows chronic lymphocyte inflammation, edema, and vascular ectasia. These pathologic changes are diagnostic of psoriasis. Also present within the dermis, there are abundant histiocytes with vacuolated cytoplasm (arrows) and large, well-defined, clear areas consistent with silicone deposits (labeled s); these pathologic changes are characteristic of a silicone granuloma. (Hematoxylin and eosin staining: A, x4; B, x10; C, x20; D, x40)

Six months after her final triamcinolone injection, a punch biopsy of the 6-mm plaque was performed. Microscopic examination of the epidermis demonstrated confluent parakeratosis with neutrophilic microabscesses, regular acanthosis, elongated rete ridges, and a thin or absent granular layer (Figure 4). The upper dermis showed chronic inflammation consisting of lymphocytes, edema, and vascular ectasia with tortuous capillaries. These pathologic findings established the diagnosis of psoriasis.

In addition, there were abundant histiocytes with vacuolated cytoplasm throughout the dermis. Stains for bacteria, fungi, and mycobacteria were negative. Correlation of the patient’s history and these pathologic findings were diagnostic of a silicone granuloma.

**Figure 5:**
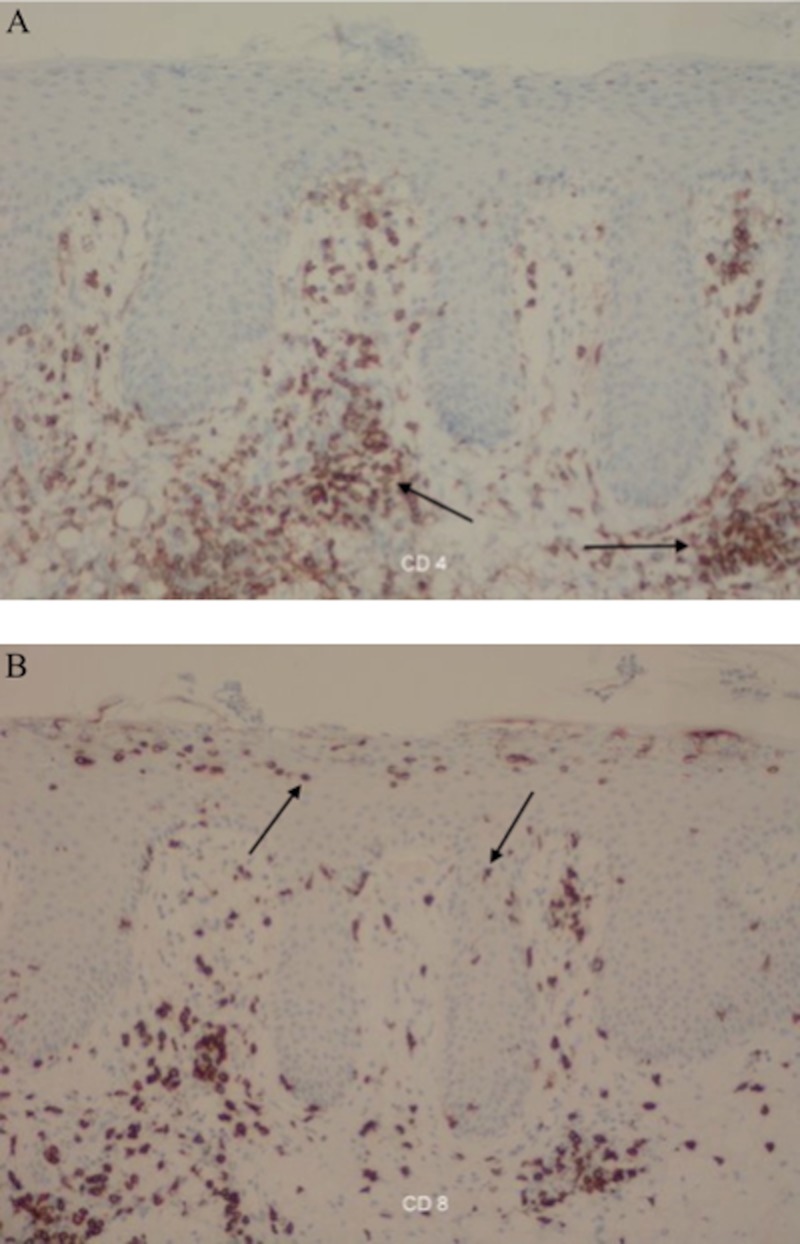
Immunoperoxidase studies of the psoriatic plaque (A) The psoriatic plaque demonstrates mononuclear cells (arrows) that are predominantly positive for cluster of differentiation 4 (CD4) in the dermis. (B) The psoriatic plaque also demonstrates mononuclear cells (arrows) that are predominantly positive for cluster of differentiation 8 (CD8) in the epidermis. (Diaminobenzidene immunoperoxidase staining: A: CD4, x20; B: CD8, x20)

Immunohistochemical studies were performed. The dermal mononuclear cells were predominantly positive for CD4 and the epidermal mononuclear cells were predominantly positive for CD8 (Figure 5). These immunohistochemistry findings are characteristic of psoriasis [4].

The entire area of psoriasis was excised in the biopsy specimen; therefore, no treatment of the lesion was required. The patient was started on oral hydroxychloroquine, 200 mg twice a day for two months, to treat the granuloma; there was no improvement. However, at her eight-month follow-up, the patient’s psoriasis lesion had not recurred and she had not developed any additional lesions of psoriasis.

## Discussion

Liquid silicone injections can cause local adverse effects at or near the site of injection; these include cellulitis, edema, granulomas, silicone migration, and ulceration [1-2]. Similarly, symptoms may occur following silicone implants, such as chronic fatigue, myalgia, and skin abnormalities [3]. Therefore, it has been proposed that silicone is capable of inducing an autoimmune/inflammatory syndrome [3].  

Psoriasiform dermatitis following liquid silicone injection has also been described in a 40-year-old man with human immunodeficiency virus (HIV) infection. His CD4 count was low, and he had an eight-month history of developing skin lesions on his buttock. The lesions were located at the site of the silicone injections and had developed after the injections. The differential diagnosis included fungal infection and HIV-associated psoriasis; biopsy confirmed the diagnosis of psoriasiform dermatitis. The investigators concluded that the psoriasiform lesions were an adverse reaction from the silicone [5].

Our patient also presented with this rare adverse effect following silicone injection. However, she was immunocompetent when her psoriatic plaque developed. Although psoriasis may occur at the site of trauma, her skin lesion developed six months after her last corticosteroid injection and several years after her silicone injections.

An immunocompromised cutaneous district is a localized skin region of immune dysregulation that becomes a vulnerable site for the development of conditions, including infection, cancer, or dermatoses, such as psoriasis [6]. Several factors can be responsible for the creation of immune dysregulation, including intradermal silicone or the granulomatous reaction to the silicone [7]. Therefore, it is reasonable to propose that the changes in our patient’s skin following her silicone injection created an immunocompromised cutaneous district; the intralesional triamcinolone acetonide may have also contributed to the development of this altered area of skin. Subsequently, the psoriatic plaque developed at this site. Indeed, psoriasis has previously been observed to occur in an immunocompromised cutaneous district [8].

## Conclusions

Psoriasis developing in an area following a liquid silicone injection in an immunocompetent patient is an unusual adverse effect. Trauma to the skin can create an immunocompromised cutaneous district; psoriasis can develop in these altered areas. Therefore, we postulate that our patient’s lesion of psoriasis resulted from either silicone independently promoting the development of psoriasis or silicone creating an immunocompromised cutaneous district, or both.
